# Clinical Features and MicroRNA Expression Patterns Between AML Patients With DNMT3A R882 and Frameshift Mutations

**DOI:** 10.3389/fonc.2019.01133

**Published:** 2019-10-24

**Authors:** Li Yang, Ke'Feng Shen, Mei'Lan Zhang, Wei Zhang, Hao'Dong Cai, Li'Man Lin, Xiao'Lu Long, Shu'Gang Xing, Yang Tang, Jie Xiong, Jia'Chen Wang, Deng'Ju Li, Jian'Feng Zhou, Min Xiao

**Affiliations:** ^1^Department of Hematology, Tongji Medical College, Tongji Hospital, Huazhong University of Science and Technology, Wuhan, China; ^2^Department of Oncology, Tongji Medical College, Tongji Hospital, Huazhong University of Science and Technology, Wuhan, China

**Keywords:** leukemia, DNMT3A gene mutations, microRNA, expression, clinical

## Abstract

**Background:** DNA methyltransferase 3A (DNMT3A) plays a unique role in hematopoiesis and acute myeloid leukemia (AML) pathogenesis. While the influences of DNMT3A mutation subtypes are still under debate.

**Purpose:** Exploration of the clinical and molecular differences between AML patients carrying DNMT3A R882 mutations and DNMT3A frameshift mutations.

**Methods:** Next generation of sequencing (NGS) and clinical data of 118 AML patients in our center were analyzed and compared. NGS, mRNA and miRNA profiling and clinical data from 12 patients in TCGA database were integrative analyzed.

**Results:** Among all patients enrolled, 113 patients were positive for the variants of interest. Overall, a total of 295 variants were discovered, among which 24 DNMT3A mutations were detected, including 1 non-sense, 20 missense, 3 frameshift mutations. And 7 DNMT3A R882 mutations (3 R882H, 2 R882C, and 2 R882P) were found. Clinical analysis from our cohort and TCGA database indicated that patients carrying DNMT3A R882 mutation exhibited significantly higher levels of peripheral blood hemoglobin and non-significantly inferior prognosis compared with patients with DNMT3A frameshift mutations. Integrative analysis indicated that miR-10b, miR-143, and miR-30a were significantly decreased in the DNMT3A R882 group. High miR-143 expression is significantly associated with better prognosis in AML patients with DNMT3A mutations.

**Conclusion:** Different molecular and clinical characteristics existed between patients with DNMT3A variant subtypes. The distinct microRNA expression pattern for DNMT3A R882 AML patients might not only act as markers to predict disease prognosis, but also could be further investigated to develop novel therapeutic targets for patients with DNMT3A mutations.

## Introduction

Acute myeloid leukemia (AML) is a kind of disease with heterogeneous pathogenic mechanisms. With the advent and progress of high-throughput sequencing techniques, the molecular landscape of AML gene mutations has been extensively investigated and uncovered (1–3). Among a series of gene mutations that present as inferior prognostic markers for AML patients, mutations in the DNMT3A gene have drawn great attention from researchers globally because these mutations play a unique role in normal hematopoiesis and in AML pathogenesis (4). DNMT3A is a kind of methyltransferase that is responsible for the *de novo* methylation of CpG dinucleotides. DNMT3A is crucial for the establishment and maintenance of cellular methylation patterns. Multiple researchers have confirmed that DNMT3A is frequently mutated in AML patients (18-23%) (1, 5, 6). The majority of the variants (approximately two-thirds of the cases) are located at R882 in exon 23. Researchers have demonstrated that the DNMT3A R882 mutation disrupts the normal ligation of methyltransferase protein subunits, causing a dominant negative impact on DNMT3A protein function (7, 8). For other non-R882 mutations, especially for DNMT3A frameshift (fs) mutations, the occurrence is much less frequent, and their significance is poorly understood. The molecular and prognostic influences of DNMT3A frameshift mutations remains obscure (9). Some studies have indicated that AML patients with DNMT3A truncating mutations have comparable prognoses to those of DNMT3A wildtype patients (10). Therefore, in this study, through the analysis of NGS sequencing data and the clinical features of AML patients at our center and in the TCGA database, we aimed to explore the molecular and biological differences between AML patients with DNMT3A frameshift mutations and those with DNMT3A R882 mutations.

## Materials and Methods

### Sample and Data Collection

Acute myeloid leukemia (AML) patients who were hospitalized in the Department of Hematology at Tongji Hospital in Wuhan, Hubei, China between February 2015 and March 2018 were retrospectively analyzed, and patients who underwent NGS analysis were enrolled. The NGS sequencing data and relevant clinical data were collected. In detail, patients' general information, AML FAB subtypes, patients' peripheral blood cell level and bone marrow blast percentage (an indicator of leukemia burden in bone marrow) from the initial diagnostic test, and chemotherapy regimen were retrieved. In addition, patients' follow-up data including personal status (dead or alive), date of last visit, and subsequent hematological stem cell transplantation therapy (HSCT) data were collected. In regard to the sample sources, 109 samples were from the bone marrow, 9 were from the peripheral blood, 1 was from subcutaneous nodules, and 1 was from the cerebral spinal fluid (CSF). For the TCGA cohort, AML patient data, including clinical information, NGS sequencing, transcriptome sequencing, and miRNA expression profiling, were retrieved from The Cancer Genome Atlas (TCGA) database (https://cancergenome.nih.gov/). This study was approved by the Ethics Review Board of Wuhan Tongji Hospital. Written informed consent was obtained for patients enrolled in our study on initial admission in our center. All the studies involving human subjects were conducted in full compliance with governmental policies and the Declaration of Helsinki. Biosample storage and experimental biosafety protocol regulation in our center were strictly followed throughout the entire study.

### Ion Torrent Next Generation Sequencing

The design of the AML sequencing panel, including 17 AML-related genes, was made using the Ion AmpliSeq™ Ready-to-Use custom designer platform (Thermo Fisher Scientific, MA, U.S.A following the website guidelines (https://www.ampliseq.com/protected/dashboard.action). Ion torrent sequencing procedures, including library preparation, emulsion PCR and sequencing, were performed as previously described (11). Raw data processing and variant calling file (VCF) generation were performed locally using the Ion Torrent platform-specific software Torrent Suite (v3.4.2 Thermo Fisher Scientific, MA, U.S.A). The Ion Torrent online annotation platform Ion Reporter (v5.10.1 Thermo Fisher Scientific, MA, U.S.A) was used for further detailed analysis of the genetic variants. All positive variants were validated with subsequent Sanger sequencing. Mutational landscape was visualized using GenVisR package from R software (version 3.1.0).

### Bioinformatics Analysis

mRNA sequencing and miRNA profiling data were retrieved from the TCGA database (https://cancergenome.nih.gov/) and were normalized using R software (version 3.1.0). Group comparisons and hierarchical clustering analysis were performed using J-Express software (Version 2012, University of Bergen, Bergen, Norway), as previously described (12). Significantly differential expressed miRNA (*p* < 0.05 by 2-way ANOVA) were included for subsequent analysis. As for the mRNA and miRNA integrative analysis, we selected genes with profoundly elevated or decreased expression (fold change >50 times or < -50 times) as potential miRNA targets. The miRTarVis+ online tool was used to identify significantly differentially expressed miRNA and target gene pairs (http://sehilyi.com/miRtarvisplus/).

### Clinical Comparison and Statistical Analysis

Group comparisons of the clinical values including WBC, Hb, Plt levels and bone marrow blast cell percentage were performed using a Student's *t*-test. All major statistical analyses were performed using SPSS 21.0 (International Business Machine Corp., Armonk, NY, USA). All *P*-values were two-sided, and the significance level was at least *P* < 0.05. The significance of gene co-mutations was compared with the χ^2^ test. Survival analysis was performed by using the Kaplan–Meier method and statistically compared by Gehan-Breslow-Wilcoxon test. OS was defined as the time from the date of diagnosis to death due to any cause.

## Results

### Mutation Spectrum in AML Patients

In this study, we performed Ion Torrent sequencing analysis of 118 patients admitted to our hospital who were diagnosed with *de novo* AML. After excluding the five patients whose variant analysis results were negative, we obtained positive AML panel sequencing results from a total of 113 patients. The patients' characteristics were shown in Supplementary Table 1.

**Figure 1:**
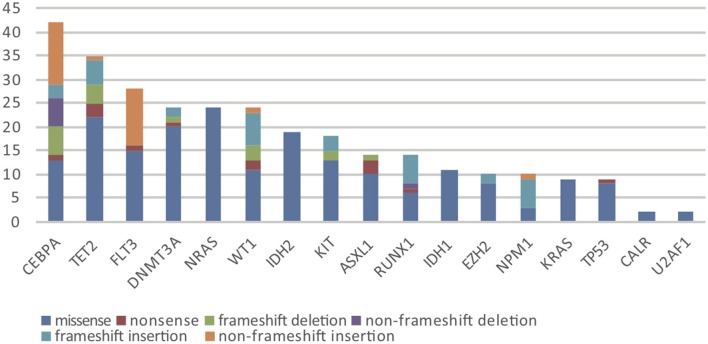
The number and subtype distribution of the 17 gene variants included in the AML NGS panel. Each variant subtype is marked with a unique color. Y axis represents the variant number (N) of each gene.

**Figure 2:**
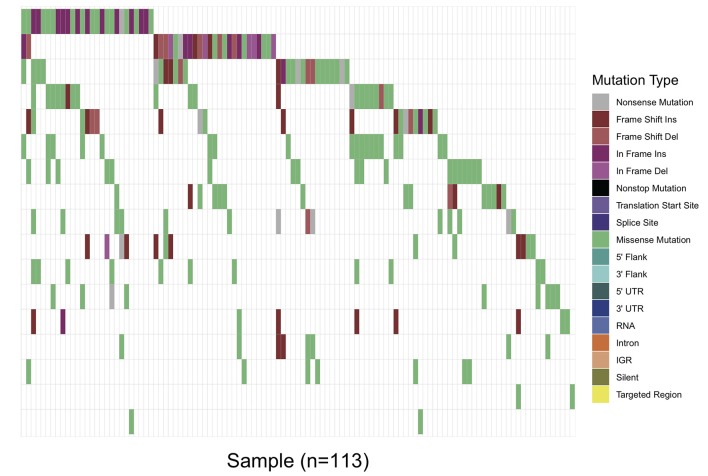
Waterfall plot of the detailed gene mutation spectrum of 113 AML patients. Each variant subtype is labeled with a unique color. The upper panel demonstrates the mutational burden per megabase DNA (MB) for each patient.

Overall, in 17 AML-related gene-coding regions, we identified a total of 295 rare variants, which were defined as gene variants with minor allele frequency (MAF) <0.01 according to previous research (11). Among them, there were 196 missense mutations, 13 non-sense mutations, 17 frameshift deletions, 7 non-frameshift deletions, 34 frameshift insertions, and 28 non-frameshift insertions. Variant-type distributions of each gene were exhibited in Figure 1 and detailed numbers of variants for each gene were listed in Supplementary Table 2. In regard to the mutation spectrum of the cohort, CEBPA was the most frequently mutated gene, which represented 14.23% of the total variants and was found in 27 patients (27/113, 23.89%). TET2 mutations were the next most frequently observed mutations, and these mutations were identified in 26 patients (26/113, 23.00%). The majority of TET2 mutations were missense variants, representing 62.85% of all TET2 variants. FLT3, with 28 positive variants among the 295 total variants (9.49%), ranked as the third most frequently mutated gene and was identified in 27 patients (27/113, 23.89%). Among these patients, the FLT3-ITD mutation was identified in 12 out of the 27 patients (44.44%), and FLT3-TKD was identified in 4 out of the 27 patients (14.81%). IDH2 mutations were less frequent and were identified in 19 patients (19/113 16.81%). Among these patients, the hotspot mutation p.R140Q was detected in 12 patients. TP53 mutations were identified in nine patients (9/113, 7.96%), among which one non-sense variant, TP53 p.Trp146Ter, was identified. In contrast, KRAS, CALR, and U2AF1 were among the genes with the lowest mutation frequencies. KRAS mutations were identified in seven patients (7/113, 6.19%). Among these patients, the p.G12D hotspot variant was found in four patients, and p.G12S was found in one patient. Detailed information on the mutation spectrum in each patient is represented as a waterfall plot (Figure 2).

### Differences in the DNMT3A Mutation Type and Clinical Characteristics of AML Patients

**Figure 3:**
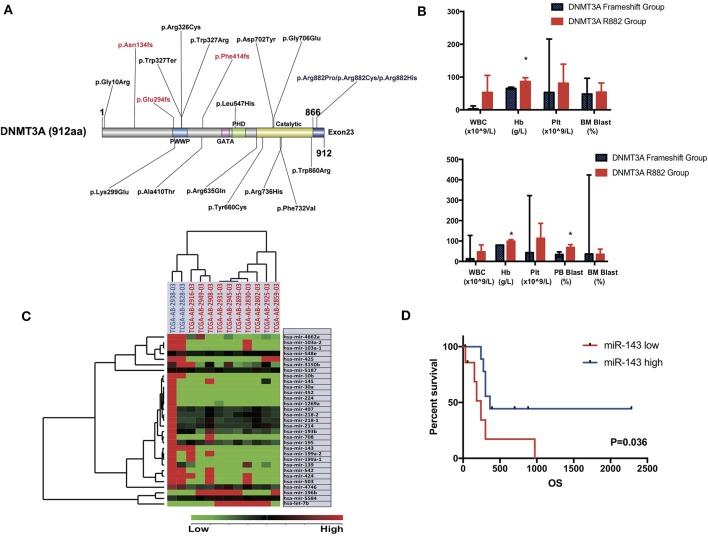
**(A)** Schematic drawing of the DNMT3A protein structure and the detailed position distribution of each variant that was detected. Each conserved domain is labeled with different colors, and three DNMT3A frameshift mutations are marked with red letters. **(B)** Bar chart of mean value and standard deviation of the clinical characteristics (WBC, hemoglobin, platelet level of peripheral blood and blast percentage in bone marrow and peripheral blood sample) of AML patients carrying DNMT3A R882 and DNMT3A frameshift mutations from the patient cohort in our center (upper chart) and TCGA cohort (lower chart). Significant differences between the two groups were compared by an unpaired *t*-test (^*^P < 0.05). **(C)** Unsupervised hierarchical clustering analysis was performed on the significantly differentially expressed microRNAs in AML cases with DNMT3A R882 and DNMT3A frameshift mutations from the TCGA database. The cluster diagram of the miRNA expression values was mean-normalized. Samples with the DNMT3A R882 mutation are labeled with a red ID marker, while those with DNMT3A frameshift mutations are labeled with a blue ID marker. **(D)** Overall survival comparison of 2 groups of AML patients carrying DNMT3A mutations (R882s and non-R882s) from the TCGA database. Patients were separated into high/low group by the expression value ranking of miR-143. Each group contained nine patients, whose expression value ranked top or bottom 45%. Survival analysis was performed with the Kaplan-Meier method and was statistically compared with the Gehan-Breslow-Wilcoxon test.

Specifically, in this study, we concentrated on the DNMT3A mutation. Overall, 24 DNMT3A variants were detected. In total, there were 20 missense variants, 1 non-sense variant, and 3 frameshift mutations. Among the 20 missense mutations, most of them located in DNMT3A conserved domains (PWWP, PHD, and catalytic domain). In detail, we identified 7 R882 mutations (3 R882H, 2 R882C, and 2 R882P) and 13 non-R882 missense variants. As for DNMT3A frameshift mutations, two of the mutations were frameshift-insertion variants (p.Asn134fs and p.Phe414fs), and one of the mutations was a frameshift-deletion variant (p.Glu294fs). Among 21 (21/113, 18.58%) patients carrying DNMT3A mutations, three were found to be simultaneously carrying two separate non-R882 DNMT3A variants. Detailed information on the 24 DNMT3A variants is summarized in Figure 3A. Regarding the co-mutated genes with DNMT3A, we found that IDH1 (*p* = 0.0159), IDH2 (*p* = 0.0004), FLT3-ITD (*p* = 0.029), and NPM1 (*p* = 0.0376) were significantly associated with DNMT3A mutations. However, in our study, FLT3-TKD was not significantly associated with the DNMT3A mutation. We next explored the differences in the clinical characteristics between AML patients carrying DNMT3A R882 and DNMT3A frameshift mutations. The clinical parameters from both groups were compared and listed in detail in Table 1. As a result, we found that the average peripheral blood hemoglobin value (Hb) in the DNMT3A R882 group was significantly higher than that in the DNMT3A frameshift group. The white blood cell (WBC), platelet (Plt), and bone marrow (BM) blast percentage were also higher in the DNMT3A R882 group (WBC 53.10^*^10^9^/L vs. 3.29^*^10^9^/L, Plt 81.4^*^10^9^/L vs. 53^*^10^9^/L, and BM blast percentage 53.9 vs. 48.4%) compared with those in the DNMT3A frameshift group (Figure 3B, upper chart). However, these differences were not statistically significant. To investigate the prognostic differences between the two groups, survival analysis was performed, and the results indicated a trend toward a longer median overall survival period (OS) in the DNMT3A frameshift group (median OS 559 days) compared with that in the DNMT3A R882 group (median OS 264 days), although the differences did not reach statistical significance (Supplementary Figure 1A). To confirm our findings, we retrieved clinical data from AML patients carrying DNMT3A mutations (10 DNMT3A R882 and 2 DNMT3A frameshift) from the TCGA database. Detailed molecular and clinical information of the patients selected were listed in Table 2. By comparing the clinical parameters between the two groups, we found that AML patients carrying DNMT3A R882 mutations also exhibited higher peripheral blood cell levels (WBC 46.9^*^10^9^/L vs. 13^*^10^9^/L, 99 g/L vs. 80 g/L, and Plt 113.2^*^10^9^/L vs. 43^*^10^9^/L) compared with those in the DNMT3A frameshift group. However, only the difference in the hemoglobin levels between the two groups reached statistical significance. Additionally, the patients with DNMT3A R882 mutations exhibited a significantly higher level of the peripheral blood blast percentage compared with that in the DNMT3A frameshift group (68.6 vs. 34, *p* = 0.035) (Figure 3B, lower chart). The results also indicated that the DNMT3A frameshift group exhibited a longer but non-significant median OS (median OS 1,294 days) compared with that in the DNMT3A R882 group (median OS 275 days) (Supplementary Figure 1B). In summary, the results from both cohorts indicated that AML patients carrying the DNMT3A R882 mutation might differ in hematopoietic functions and in disease prognosis compared with those of the patients in the DNMT3A frameshift group.

**Table 1:** Detailed molecular and clinical characteristics of AML patients carrying DNMT3A R882 and DNMT3A frameshift mutations from the cohort at our center.

**Group**	**DNMT3A variant type**	**Combing mutations**	**DNMT3A variant frequency**	**AML type**	**Cytogenetic**	**Age**	**Gender**	**WBC count**	Hb^*^	**Plt**	**BM blasts %**	**HSCT**
Frameshift	p.Phe414fs	IDH1 p.Lys27Gln FLT3-ITD	0.453	M2/M4	Not available	64	F	7.38	66	25	34.60	NO
Frameshift	p.Glu294fs	IDH2 p.Arg172Lys	0.257	M5	+4,+6,+20	42	F	1.12	67	128	70.50	NO
Frameshift	p.Asn134fs	EZH2 p.Arg213His /ASXL1 p.Gln561Ter/TET2 p.Leu1329Val	0.132	M2	*t*_(8, 21)_	39	M	1.38	64	6	40	NO
SNV	p.Arg882Pro	FLT3 p.Asn609Tyr/ASXL1 p.Ile268Val /	0.527	M5	11q+	54	F	161.8	81	50	86	NO
SNV	p.Arg882Cys	NRAS p.Gln61Leu/p.Gly13Asp	0.305	M5	Normal	64	M	40.75	74	54	72.50	NO
SNV	p.Arg882His	IDH2 p.Arg140Gln	0.280	M4	Normal	65	M	15.6	105	165	27.50	NO
SNV	p.Arg882Cys	IDH1 p.Arg132His/FLT3 p.Arg595_Glu596ins13/WT1 p.Glu47Lys/TET2 p.His1550Gln/ASXL1 p.Leu1266Val/NPM1 p.Trp288fs	0.516	M5b	Normal	37	F	81.05	70	176	96.50	NO
SNV	p.Arg882His	IDH1 p.Arg132His/NRAS p.Gly12Asp/NRAS p.Gly13Val/IDH2 p.Met397Val/NPM1 p.Trp288fs	0.453	M1	Normal	51	F	63.55	90	30	40	NO
SNV	p.Arg882His	RUNX1 p.Ala142fs/CEBPA p.Asn74fs/TET2 p.Glu100Ter	0.398	M2	+8	41	M	3.32	92	25	27	YES
SNV	p.Arg882Pro	None	0.347	M5b	Normal	54	M	5.82	95	70	28	YES

Significant differences between the two groups were compared by an unpaired t-test (

* *P < 0.05)*.

**Table 2:** Detailed molecular and clinical characteristics of AML patients carrying DNMT3A R882 and DNMT3A frameshift mutations from a cohort in the TCGA database.

**Group**	**Tumor sample**	**Combining mutations**	**FAB type**	**Cytogenetic**	**Age (y)**	**WBC (10^**∧**^9/L)**	Hb (g/L)^*^	**Plt (10^**∧**^9/L)**	PB blast percent (%)^*^	**BM blast percent (%)**
Frameshift	TCGA-AB-2938-03A-01W-0732-08	FLT3 Mutation Negative|IDH1 R132 Negative|IDH1 R140 Negative|IDH1 R172 Negative|Activating RAS Negative|NPMc Negative	M7	Intermediate/ Normal	76	4	8	21	33	6
Frameshift	TCGA-AB-2828-03B-01W-0728-08	FLT3 Mutation Negative|IDH1 R132 Negative|IDH1 R140 Negative|IDH1 R172 Negative|Activating RAS Negative|NPMc Negative	M4	Favorable	55	22	8	65	35	67
R882	TCGA-AB-2931-03A-01W-0745-08	PML-RAR Negative|FLT3 Mutation Negative|IDH1 R132 Positive|IDH1 R140 Negative|IDH1 R172 Negative|Activating RAS Negative|NPMc Negative	M4	Intermediate/ Normal	50	17	10	231	88	52
R882	TCGA-AB-2916-03A-01W-0732-08	FLT3 Mutation Negative|IDH1 R132 Negative|IDH1 R140 Negative|IDH1 R172 Negative|Activating RAS Negative|NPMc Negative	M4	Intermediate/ Normal	64	3	11	134	85	22
R882	TCGA-AB-2895-03A-01W-0733-08	FLT3 Mutation Negative|IDH1 R132 Negative|IDH1 R140 Negative|IDH1 R172 Negative|Activating RAS Negative|NPMc Positive	M2	Intermediate/ Normal	40	8	10	95	47	23
R882	TCGA-AB-2949-03A-01W-0733-08	FLT3 Mutation Positive|IDH1 R132 Negative|IDH1 R140 Negative|IDH1 R172 Negative|Activating RAS Negative|NPMc Positive	M1	[Not Available]	41	134	12	40	92	90
R882	TCGA-AB-2859-03B-01W-0728-08	FLT3 Mutation Negative|IDH1 R132 Negative|IDH1 R140 Negative|IDH1 R172 Negative|Activating RAS Negative|NPMc Negative	M2	Poor	81	2	10	39	46	0
R882	TCGA-AB-2945-03A-01W-0733-08	FLT3 Mutation Negative|IDH1 R132 Negative|IDH1 R140 Negative|IDH1 R172 Negative|Activating RAS Negative|NPMc Negative	M4	Intermediate/ Normal	48	12	9	351	51	5
R882	TCGA-AB-2908-03A-01W-0745-08	BCR-ABL Negative|FLT3 Mutation Positive|IDH1 R132 Negative|IDH1 R140 Negative|IDH1 R172 Negative|Activating RAS Negative|NPMc Positive	M5	Intermediate/ Normal	57	99	10	80	52	4
R882	TCGA-AB-2802-03B-01W-0728-08	FLT3 Mutation Positive|IDH1 R132 Negative|IDH1 R140 Negative|IDH1 R172 Negative|Activating RAS Negative|NPMc Negative	M2	Intermediate/ Normal	75	98	8	31	76	80
R882	TCGA-AB-2925-03A-01W-0732-08	FLT3 Mutation Positive|IDH1 R132 Positive|IDH1 R140 Negative|IDH1 R172 Negative|Activating RAS Negative|NPMc Positive	M4	Poor	65	49	9	76	90	0
R882	TCGA-AB-2830-03B-01W-0728-08	FLT3 Mutation Negative|IDH1 R132 Positive|IDH1 R140 Negative|IDH1 R172 Negative|Activating RAS Negative|NPMc Negative	M0 Undifferentiated	Poor	58	47	10	55	59	72

Significant differences between the two groups were compared by an unpaired t-test (

* *P < 0.05)*.

### miRNA-mRNA Integrative Analysis Indicated That the DNMT3A R882 Mutation Group Exhibited a Distinct miRNA Expression Pattern

To further explore the underlying mechanism of the clinical and prognostic differences between AML patients carrying DNMT3A R882 and DNMT3A frameshift mutations, we retrieved the mRNA sequencing and miRNA profiling data from 12 AML patients from the TCGA database, as described above. The miRTarVis+ online tool was used to perform an integrative analysis of the significantly differentially expressed miRNAs and their target genes. The miRNA and transcriptome data was processed according to previous research (13). As a result, we identified 31 significantly differentially expressed miRNAs (*p* < 0.01) based on 2-way ANOVA and hierarchical clustering (Figure 3C). mRNA sequencing demonstrated that a group of HOX family genes were upregulated in the DNMT3A R882 group compared with those in the DNMT3A frameshift group. Further, through combined analysis with the mRNA sequencing results, three miRNAs attracted our attention, as they were significantly decreased in the DNMT3A R882 group compared with those in the DNMT3A frameshift group, as shown in Table 3. Specifically, compared to those in the frameshift group, miR-10b exhibited significantly decreased expression (fold change 18.26, *p* = 0.002) in the DNMT3A R882 group, while its target HOXA3 exhibited increased expression (fold change 146.13). Compared to those in the frameshift group, miR-143 exhibited a 5.83-fold decrease in expression (*p* = 0.008) in the DNMT3A R882 group, while its target, HOXA7, was upregulated (fold change 176.09). Additionally, compared to those in the frameshift group, miR-30a exhibited significantly decreased expression (fold change 17.56, *p* = 0.009) in the DNMT3A R882 group, while its target HOXA11 was upregulated (fold change 345.68).

**Table 3:** miRTarVis+ integrative mRNA-miRNA analysis of three significantly differentially expressed miRNAs and the fold change of the corresponding target mRNA.

**miRNA**	**miRNA *P*-value**	**miRNA fold change**	**Target gene**	**Target gene *P*-value**	**Target gene fold change**
hsa-mir-10b	0.0020303	−18.26049	HOXA3	0.1558089	146.138004
hsa-mir-143	0.008674014	−5.838354	HOXA7	0.1180796	176.0971778
hsa-mir-30a	0.009734094	−17.56989	HOXA11	0.3178429	345.6827711

*P-values were calculated with 2-way ANOVA*.

In order to evaluated the prognostic impact of these microRNAs. We separated AML patients carrying DNMT3A mutations (R882 and non-R882) from TCGA database into two groups according to the expression value ranking of three microRNAs. In detail, patients with miRNA expression ranking top 45% among all were selected in miRNA high group and those with bottom 45% were selected in miRNA low group, respectively. Prior co-mutated genes analysis demonstrated no significant differences in patients' number with DNMT3A R882, FLT3-ITD, IDH1, or NPM1 mutations between two groups. Result indicated that patients in miR-143 high group exhibited significantly better prognosis compared with those in miR-143 low group (*p* = 0.036, Gehan-Breslow-Wilcoxon test) (Figure 3D). However, no significant prognostic differences were identified between miR-10b high/low or miR-30a high/low group (Supplementary Figures 1C,D).

## Discussion

Generally, in this study, the mutation frequency of FLT3, CEBPA, IDH1/2, RAS, and TP53 detected in our center was comparable to those in previous publications (3, 14, 15). However, the frequency of TET2 variants was much higher which could be explained by the unique age distribution in our cohort. Current studies have indicated that TET2 mutations occur in adult AML patients with a mutation frequency between 7%-10%, while in older patients (>60 years), the TET2 mutation rate could reach 19%-24% (6, 16, 17). Moreover, the mutation frequency of DNMT3A and NPM1 was lower than those in previous reports, which was partially due to the number of pediatric patients enrolled in our cohort. It has been reported that DNMT3A and NPM1 were significantly less frequently mutated in pediatric AML patients (18–20) than in adult AML patients.

Previous studies have demonstrated that R882 variants make up 40–60% of all DNMT3A mutations (21). The lower percentage of R882 variants in our cohort could have been caused by the bias due to the limited sample size. In regard to the genes that were co-mutated with DNMT3A, we demonstrated that IDH1/IDH2, NPM1, FLT3-ITD mutations were significantly associated with DNMT3A mutations, which was in accordance with previous reports (22, 23).

As for the clinical impact of DNMT3A mutation subtypes, up till now, there has been limited evidence on the clinical impact of the different DNMT3A mutation types in AML patients. One large cohort study from the United Kingdom demonstrated that DNMT3A R882 patients had significantly higher WBC level than that of DNMT3A non-R882 patients (10). In addition, for DNMT3A non-R882 patients, those with missense mutations had a higher median WBC than those with truncation mutations. However, another large cohort study indicated that no significant differences were found in clinical characteristics between patients with DNMT3A R882 and non-R882 mutations (4). The results from our study indicated that AML patients in the DNMT3A R882 group exhibited higher WBC, Hb, and Plt levels compared with those in the DNMT3A frameshift group. However, only the Hb level difference between two cohorts reached statistical significance.

Additionally, data from the TCGA cohort indicated that the DNMT3A R882 group exhibited significantly increased percentage of peripheral blood leukemic blast compared with DNMT3A frameshift group. This result is in accordance with previous research (4), which is also evidence of increased proliferative capability of leukemic stem cells carrying DNMT3A R882 mutations. Unfortunately, we could not validate this result in our cohort due to lack of data.

As for the prognostic impact, due to the very limited sample size, only non-significantly longer OS was observed in the DNMT3A frameshift group compared with that in the DNMT3A R882 group. Current opinions differ on the prognostic impact between the two mutation groups (4, 10). The AMLSG study indicated that DNMT3A R882 mutation presented as an inferior prognostic marker, while DNMT3A non-R882 mutations presented as a better prognostic marker (4). Meanwhile, another study demonstrated that patients with DNMT3A R882 had a shorter OS than that of patients with DNMT3A wildtype status, the survival of the patients with DNMT3A truncation mutations was similar to that of DNMT3A wildtype patients (10). Therefore, studies with a larger cohort from multiple centers are still of great importance to determine the exact effects of the different DNMT3A mutation subtypes on AML patients' clinical characteristics.

As for the molecular impact, previous molecular studies have indicated that DNMT3A truncating mutations generally result in non-sense decay and in haploinsufficiency, and the transplantation of stem cells with DNMT3A deletion did not cause apparent changes in hematopoiesis in mouse models (24, 25). On the other hand, DNMT3A R882 generally had dominant-negative effects, as mutated DNMT3A proteins hamper the formation of functional homotetramers (8). A previous methylation study found that focal hypomethylation at specific CpG residues in DNMT3A R882 mutated patients was distinct from that in non–R882-mutated patients (26). However, the exact molecular mechanism through which DNMT3A R882 causes increased peripheral blood cell levels remains obscure, although some researchers have hypothesized that the phenomenon could be explained by the significantly associated co-mutations, FLT3-ITD, and NPM1 (4). Evidences also indicated that increased HOX family gene expression in DNMT3A mutated AML patients resulted in enhanced myelopoiesis (27–29). The above findings did help to explain the different impact of DNMT3A R882 and DNMT3A frameshift mutations.

However, whether DNMT3A mutation subtypes influence AML phenotype through modulation of miRNA profile is largely unknown. In our study, three differentially expressed miRNAs caught our attention. All three miRNAs target the HOX gene family members, which were upregulated in the DNMT3A R882 group compared to that in the DNMT3A frameshift group. In detail, miR-10b belongs to the miR-10 family, and miR-10b acts as a novel oncogene in many types of cancer (30). Recent studies demonstrated that miR-10 family regulated multiple tumor cell functions by targeting HOX family genes (31–33). miR-10b was also shown to be significantly upregulated in NPM1-mutated AML patients (34). A cell model study indicated that miR-10b might result in unlimited proliferation of immature blood progenitors and in the repression of mature blood cell differentiation and maturation, thus leading to the occurrence of AML (30). miR-143, on the other hand, has been suggested to be a tumor suppressor, and significantly decreased level of miR-143 was detected in AML patients compared to those in healthy controls (35). Moreover, miR-143 may regulate epigenetic modification by silencing DNMT3A expression (36). miR-30a was found to be abnormally decreased in MDS patient samples (37), but current studies have not confirmed its influence on AML pathogenesis.

To further investigate the prognostic prediction value of these three miRNAs, the survival analysis from our study did support the theory that miR-143 high expression played a protective role for AML patients with the presence of DNMT3A mutations. Unfortunately, in our study we failed to identify significant prognostic difference between miR-10b, miR-30a high/low group. However, this conclusion requires further confirmation in an expanded cohort study. Therefore, it would be of importance if miR-143 were validated as predictive markers for AML patients with DNMT3A patients. Patients with miR-143 low expression might require more intensive therapeutic strategy such as HSCT. Finally, the exact role of these microRNAs playing in AML phenotype, especially for patients with DNMT3A R882 mutations, requires more thorough functional research.

## Conclusion

In summary, in this study, we retrospectively analyzed the next-generation sequencing data from AML patients in our center. Through a combinatory study of the clinical characteristics and sequencing results from two independent cohorts, we demonstrated that patients with different DNMT3A mutation types exhibited varied clinical features and disease prognoses. Further detailed analysis indicated that for AML patients, different DNMT3A mutation types were related to unique miRNA expression patterns. These differentially expressed miRNAs could not only act as markers to predict disease prognosis, but also be further investigated to develop novel therapeutic targets for patients with DNMT3A mutations.

## Data Availability Statement

Our study focused on the sequencing data from TCGA database, in project TCGA-LAML, with DbGaP Study Accession number phs000178.

## Ethics Statement

The studies involving human participants were reviewed and approved by Ethics Review Board of Wuhan Tongji Hospital. Written informed consent to participate in this study was provided by the participants' legal guardian/next of kin.

## Author Contributions

LY designed the study and wrote the manuscript. KS and MZ performed the NGS experiments. WZ and HC performed bioinformatic analysis. LL, XL, SX, and YT contributed in the clinical information gathering and analysis. JX and JW contributed in the public database search and TCGA analysis. DL, JZ, and MX supervised and coordinated the whole research.

### Conflict of Interest

The authors declare that the research was conducted in the absence of any commercial or financial relationships that could be construed as a potential conflict of interest.
