# Supplementary material for: Clinical Features and MicroRNA Expression Patterns Between AML Patients With DNMT3A R882 and Frameshift Mutations
Source: Front Oncol. 2019 Oct 24;9:1133. doi: 10.3389/fonc.2019.01133 (PMC6821681; doi:10.3389/fonc.2019.01133)
Supplement: Supplementary file 3 [file Data_Sheet_1.docx]

**Supplementary table legends**

**Supplementary table 1**

Patients’ general characteristics including age, sex, and disease FAB-subtypes distribution of 113 patients with positive NGS result in our center

**Supplementary table 2**

Detailed variant numbers and subtype distributions of the 17 genes included in the AML NGS panel.

**Supplementary Figure Legend**

**Supplementary Figure 1**

1. Overall survival comparison of AML patients carrying DNMT3A R882 and DNMT3A frameshift mutations from the cohort at our center. Survival analysis was performed with the Kaplan-Meier method and was statistically compared with the Gehan-Breslow-Wilcoxon test.
2. Overall survival comparison of AML patients carrying DNMT3A R882 and DNMT3A frameshift mutations from the cohort from TCGA database. Survival analysis was performed with the Kaplan-Meier method and was statistically compared with the Gehan-Breslow-Wilcoxon test.
3. - D)

Overall survival comparison of 2 groups of AML patients carrying DNMT3A mutations (R882s and non-R882s) from the TCGA database. Patients were separated into high/low group by the expression value ranking of miR-10b and miR-30a . Each group contained patients whose expression value ranked top or bottom 45%. Survival analysis was performed with the Kaplan-Meier method and was statistically compared with the Gehan-Breslow-Wilcoxon test.
