# Supplementary figures and images for: Clinical Features and MicroRNA Expression Patterns Between AML Patients With DNMT3A R882 and Frameshift Mutations
Source: Front Oncol. 2019 Oct 24;9:1133. doi: 10.3389/fonc.2019.01133 (PMC6821681; doi:10.3389/fonc.2019.01133)

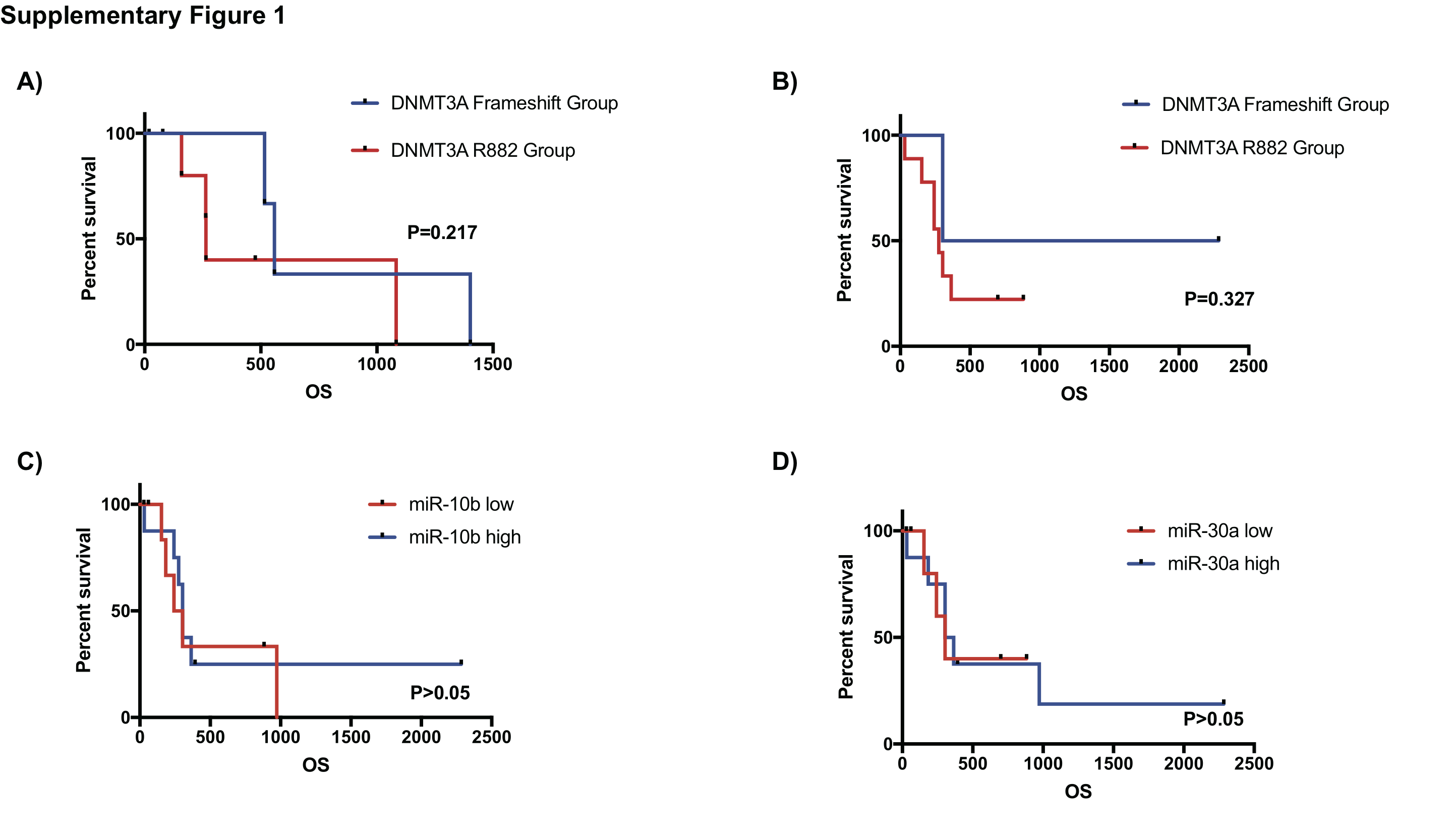

Supplement: Supplementary file 4 [file Image_1.TIFF]
